# Focal Geometry and Characteristics of Erosion-Prone Coronary Plaques *in vivo* Angiography and Optical Coherence Tomography Study

**DOI:** 10.3389/fcvm.2021.709480

**Published:** 2021-09-08

**Authors:** Muhua Cao, Tianyu Wu, Jiawei Zhao, Zhuo Du, Zhuozhong Wang, Lulu Li, Guo Wei, Jinwei Tian, Haibo Jia, Gary S. Mintz, Bo Yu

**Affiliations:** ^1^Department of Cardiology, The Second Affiliated Hospital of Harbin Medical University, Harbin, China; ^2^The Key Laboratory of Myocardial Ischemia, Chinese Ministry of Education, Harbin, China; ^3^Cardiovascular Research Foundation, New York, NY, United States

**Keywords:** erosion-prone plaque, predictors, plaque erosion, optical coherence tomography, acute coronary syndrome

## Abstract

**Objective:** This study compared focal geometry and characteristics of culprit plaque erosion (PE) vs. non-culprit plaques in ST-segment elevated myocardial infarction (STEMI) patients in whom optical coherence tomography (OCT) identified PE as the cause of the acute event.

**Background:** Culprit PE is a distinct clinical entity with specific coronary risk factors and its own tailored management strategy. However, not all plaques develop erosion resulting in occlusive thrombus formation.

**Methods:** Between January 2017 and July 2019, there were 484 STEMI patients in whom OCT at the time of primary percutaneous intervention identified culprit lesion PE to be the cause of the event; 484 culprit PE were compared to 1,132 non-culprit plaques within 1,196 imaged vessels.

**Results:** Culprit PE were highly populated at “hot spots” within the proximal 40 mm in the left anterior descending artery (LAD) and tended to cluster proximal to a nearby bifurcation mainly in the LAD. Minimal lumen area (MLA) <2.51 mm^2^ and AS (area stenosis) >64.02% discriminated culprit PE from non-culprit plaques. In the multivariable analysis, focal geometry (LAD location, distance from coronary ostium <40 mm, and location proximal to a nearby bifurcation), luminal narrowing (MLA <2.51 mm^2^, AS > 64.02%), and TCFA phenotype were independent predictors of culprit PE overall. Cholesterol crystals were predictive of culprit PE with underlying LRP morphology while the absence of calcification and microchannels were risk factors for culprit PE with an underlying non-LRP. Similarities and differences in predictors of culprit PE were found between males and females; distance from coronary ostium <40 mm, MLA <2.51 mm^2^, TCFA, and less spotty calcium were risk factors of culprit PE in males, but not in females while smaller RVD was associated with culprit PE only in females.

**Conclusions:** Irrespective of underlying lesion substrates and patient risk factors, there are lesion-specific and OCT-identifiable predictors of developing culprit PE in erosion-prone vulnerable patients.

## Introduction

Plaque erosion (PE) is a distinct pathological and clinical entity and the second most common cause of coronary thrombosis; it is responsible for 25–35% of acute coronary syndrome (ACS) and may have its own tailored management strategy (1). Furthermore, patients with culprit eroded plaques have a lower prevalence of rupture-related pancoronary, non-culprit lesion instability to include a lower prevalence of thin-cap fibroatheromas (TCFA), plaque ruptures, and high-risk plaques as defined in the CLIMA study (NCT02883088), regardless of systemic risk factor profiles (2).

Features of PE include detachment of the endothelium and platelet activation and aggregation. Flow disturbances appear first leading to chronic and persistent endothelial activation and injury (3). However, not all plaques in an erosion-prone vulnerable patient develop into erosions resulting in occlusive thrombus formation, suggesting that certain plaques may be at increased risk by virtue of their particular plaque features and focal geometry.

While there is ample evidence for risk factor predictors of an erosion-prone vulnerable patient (4–7), there is a scarcity of *in vivo* data regarding lesion-specific predictors for erosion-prone plaques within an erosion-prone vulnerable patient. Therefore, the present study compared plaque characteristics and focal geometry of culprit PE vs. non-culprit plaques in a large series of ST-segment elevation myocardial infarction (STEMI) patients in whom optical coherence tomography (OCT) identified plaque erosions as the cause of the acute event.

## Materials and Methods

### Study Population

Between January 2017 and July 2019, 2,136 patients (≥18 years of age) presenting with STEMI were treated emergently with OCT imaging in the Cardiovascular Hospital of the 2nd Affiliated Hospital of Harbin Medical University (Harbin, China). Criteria for the diagnosis of STEMI have been described previously (8). Patients with OCT imaging of the culprit vessel after pre-dilation (*n* = 28), who presented with in-stent restenosis or thrombosis (*n* = 66), or with suboptimal image quality or very short analyzable segment (*n* = 81) or incomplete demographic or clinical or imaging data (*n* = 30) were excluded. After excluding STEMIs caused by culprit plaque rupture, calcified nodule, and other culprit plaque phenotypes, 484 STEMI patients in whom the acute event was caused by culprit PE as identified by OCT imaging were included in the present study (Supplementary Figure 1).

Manual thrombectomy was performed in the setting of initial of thrombolysis in myocardial infarction (TIMI) flow grade ≤ 1 or extensive thrombus. OCT of the culprit artery was performed before percutaneous coronary intervention (PCI) while OCT of the non-culprit arteries was performed after the culprit lesion was treated. Accordingly, 1,132 non-culprit plaques were identified within 1,196 imaged vessels (single-, double-, and triple-vessel OCT imaging in 71, 114, and 299, respectively). Criteria for traditional risk factors have been included in the Supplementary Materials. The present study complied with the Declaration of Helsinki and was approved by the Ethics Committee of the 2nd Affiliated Hospital of Harbin Medical University, and all patients provided written informed consent.

### Coronary Angiography Analysis

Quantitative coronary angiography (QCA) analysis was performed using Cardiovascular Angiography Analysis System (CAAS, 5.10, Pie Medical Imaging B.V., Maastricht, The Netherlands). Coronary flow was assessed with the TIMI flow grade classification. QCA parameters including the reference vessel diameter (RVD), minimal lumen diameter (MLD), diameter stenosis (DS), and lesion length were measured post-thrombectomy from end-diastolic frames and calibration using the catheter tip (9). The distance from culprit or non-culprit lesions to the respective coronary ostium was measured in a non-foreshortened view (9). Lesions assessed angiographically were matched to OCT using fiduciary sidebranches.

### OCT Image Acquisition and Analysis

OCT imaging was acquired with a commercially available C7-XR or ILUMIEN OPTIS or OPTIS Integrated System (Abbott Vascular, Santa Clara, CA, USA) (2, 10, 11). As noted above, pre-intervention OCT of the culprit lesion was performed before and OCT imaging of non-culprits was performed after treatment of the infarct lesion. In case of long vessel segments, imaging was performed using multiple pullbacks that were then “stitched” together and overlapped to assess the entire vessel. OCT was performed in the mid or distal segments in most studied vessels (88.2% of left anterior descending artery [LAD]; 84.7% of right coronary artery [RCA] and 64.4% of left circumflex artery [LCX]). The total length of analyzed OCT pullbacks was 206.3 ± 35.5 mm (70.8 ± 25.5 in the LAD; 84.1 ± 19.1 in the RCA and 45.8 ± 15.5 in the LCX) (Supplementary Table 1).

All OCT images were submitted for core laboratory analyses that were carried out by two independent investigators (M.C. and T.W.) who were blinded to clinical, angiographic, and laboratory data using an offline review workstation (Abbott Vascular) (2, 10, 11). Any discordance was resolved by consensus with a third reviewer (Z.D.). Quantitative and qualitative analyses of all lesions were performed as previously described and as presented in the Supplementary Material (2, 8, 11, 12). Culprit lesions were identified based on angiographic findings, ECG changes, and/or left ventricular wall motion abnormalities (8). All plaques were identified by OCT as segments with a loss of the normal three-layered structure of the vessel wall. At least three consecutive 1 mm cross-sections with these features were necessary to define a plaque (2, 11, 12). A distance of at least 5 mm on the longitudinal view was necessary to consider two plaques as separate (12).

Based on established OCT diagnostic criteria, PE was identified by the presence of attached thrombus overlying an intact and visualized plaque, luminal surface irregularity at the culprit lesion in the absence of thrombus, or attenuation of the underlying plaque by thrombus without superficial lipid or calcification immediately proximal or distal to the site of thrombus (Supplementary Figure 2) (8). Excellent intra-observer and inter-observer agreement was observed in the identification of culprit PE (κ, 0.93 and 0.89, respectively).

Quantitative analysis was performed at 1 mm intervals on cross-sectional OCT images. Proximal and distal references were the sites with the largest lumen area proximal and distal to the lesion, but within the same segment; and a mean reference lumen area was calculated. Minimal lumen area (MLA) was the smallest lumen area within the length of the lesion. For culprit PE and non-culprit plaque with thrombus, MLA was estimated excluding the thrombus and was used to determine luminal percent stenosis of the pre-thrombotic plaque [100 × (1-MLA/mean of reference areas)]. The method for tracing lumen area and MLA has been presented in Supplementary Figure 3. For lesions without thrombus, percent area stenosis was calculated as [100 × (1-MLA/mean of reference areas)]. Minimal flow area (MFA) was the smallest flow area within the length of the lesion (13). Percent area stenosis (AS) was calculated as (([Mean Reference Lumen Area-MLA]/Mean Reference Lumen Area) ×100).

The distance from each lesion to a nearby bifurcation was determined from the pre-thrombotic MLA site to the bifurcation (Supplementary Figure 3). “Nearby bifurcation” was a sidebranch (with an orifice diameter >1.0 mm measured by OCT) within 5 mm proximal or distal to the lesion (4).

### Statistical Methods

Data distribution was assessed according to the Kolmogorov-Smirnov test. Continuous variables were expressed as mean ± standard deviation or median (interquartile range), and compared using the independent samples Student's test or Mann-Whitney U test. Categorical data were presented as counts (proportions) and were compared using the Chi-square or Fisher's exact test. Receiver-operating characteristic (ROC) analysis and calculation of sensitivity and specificity were performed to test the ability of MLA and AS to differentiate culprit PE from non-culprit plaques. Comparisons of per-lesion data were performed using the generalized estimating equations (GEE) to take into account potential clustering of multiple plaques in a single patient. Predictors of culprit PE were analyzed by the multi-variable logistic regression model with GEE. Variables tested included location in the LAD, distance from coronary ostium <40 mm, location proximal to a nearby bifurcation, MLA <2.51 mm^2^, AS >64.02%, RVD, lipid rich plaque (LRP), TCFA, cholesterol crystals, macrophages, calcification, spotty calcium, and microchannels. Variables that showed *P* <0.10 in the univariate model were entered into the multivariate model. Intra-observer and inter-observer differences were quantified using the κ coefficient of agreement for the culprit plaque identification. A two-sided *P* <0.05 was considered statistically significant. Statistical analyses were performed using SPSS version 23.0 (IBM Corp, Armonk, NY).

## Results

### Patient Characteristics

Baseline characteristics and laboratory findings of STEMI patients with culprit PE have been shown in Table 1.

**Table 1:** Baseline characteristics and laboratory findings of STEMI patients with culprit plaque erosion.

**Variables**	**Patients with culprit PE (*n* = 484)**
Male	364 (75.2)
Age (years)	55.2 ± 11.6
Age <50 years	165 (34.1)
**Risk factors**
Diabetes mellitus	82 (16.9)
Dyslipidemia	243/462 (52.6)
Current smoker	281 (58.1)
Hypertension	203 (41.9)
CKD	42/483 (8.7)
**Laboratory data**
TC (mg/dL)	178.1 ± 40.6
Triglyceride (mg/dL)	118.3 (83.4–179.1)
LDL-C (mg/dL)	108.9 ± 32.5
HDL-C (mg/dL)	50.1 ± 12.2
TC/HDL ratio	3.7 ± 1.2
HbAlc (%)	5.7 (5.4–6.1)
hs-CRP (mg/L)	4.5 (2.1–10.6)
Hemoglobin (g/L)	148.2 ± 20.0
**Previous history**
Previous MI	5 (1.0)
Previous PCI	1 (0.2)

*Values shown are n (%), mean ± SD, or median (25th−75th percentiles). CKD, chronic kidney disease; HDL-C, high-density lipoprotein cholesterol; hs-CRP, high-sensitive C-reactive protein; LDL-C, low-density lipoprotein cholesterol; MI, myocardial infarction; PCI, percutaneous coronary intervention; PE, plaque erosion; STEMI, ST-segment elevation myocardial infarction; TC, total cholesterol*.

### Angiographic Findings

QCA results including RVD, MLD, DS, and lesion length comparing culprit PE to non-culprit plaques have been presented in Table 2. Culprit PE were longer and had more severe diameter stenosis than non-culprit plaques.

**Table 2:** QCA and OCT analysis of culprit and non-culprit plaques in patients with culprit plaque erosion.

**Variables**	**Culprit PE (*n =* 484)**	**Non-culprit plaque (*n =* 1,132)**	***P*-value**
**QCA analysis**
RVD (mm)	3.02 ± 0.63	2.92 ± 0.66	0.519
MLD (mm)	1.13 ± 0.43	1.70 ± 1.21	<0.001
DS (%)	63.4 (54.5–70.6)	42.0 (35.1–50.0)	<0.001
Lesion length (mm)	13.8 (10.4–18.0)	9.7 (7.4–13.3)	<0.001
**OCT analysis**
Lesion length (mm)	19.9 ± 5.9	14.6 ± 6.0	<0.001
Proximal reference lumen area (mm^2^)	8.7 ± 3.4	8.3 ± 3.3	0.682
Distal reference lumen area (mm^2^)	7.1 ± 3.3	7.4 ± 3.3	0.238
MLA (mm^2^)	2.2 ± 1.4	3.8 ± 2.0	<0.001
MFA (mm^2^)	1.6 ± 1.2	3.8 ± 2.1	<0.001
AS (%)	72.0 ± 13.7	53.2 ± 14.2	<0.001
**Lipid rich plaques**
Thinnest FCT (μm)	70.7 ± 32.7	84.8 ± 37.9	0.054
Mean lipid arc (°)	213.5 ± 61.5	163.3 ± 47.2	<0.001
Maximum lipid arc (°)	285.3 ± 80.9	205.4 ± 72.6	<0.001
Lipid core length (mm)	5.5 (3.2–8.5)	3.6 (2.0–6.1)	0.240
Lipid index	1105.2 (612.8–1812.1)	587.0 (281.5–1016.3)	0.008
TCFA	126 (26.0)	135 (11.9)	<0.001
Cholesterol crystal	169 (34.9)	189 (16.7)	0.055
Macrophage	302 (62.4)	608 (53.7)	0.124
Microchannel	97 (20.0)	312 (27.6)	0.512
Spotty calcium	110 (22.7)	276 (24.4)	0.103
Calcification	185 (38.2)	409 (36.1)	0.216
Calcification length	3.9 (2.1–7.9)	3.4 (1.7–6.4)	0.005
Mean calcification arc	77.1 ± 31.9	67.4 ± 33.0	0.004
Max calcification arc	109.3 (73.2–164.0)	88.6 (58.2–124.9)	<0.001
Calcification index	292.0 (122.0–614.9)	209.1 (91.2–447.0)	<0.001
Thrombus	436 (90.1)	49 (4.3)	<0.001
White	389 (89.2)	43 (87.8)	0.755
Red	47 (10.8)	6 (12.2)	

*Values shown are n (%), mean ± SD, or median (25th−75th percentiles). AS, Area stenosis; DS, diameter stenosis; FCT, fibrous-cap thickness; MFA, minimal flow area; MLA, minimal lumen area; MLD, minimal lumen diameter; OCT, optical coherence tomography; PE, plaque erosion; QCA, quantitative coronary angiography analysis; RVD, reference vessel diameter; TCFA, thin-cap fibroatheroma*.

### OCT Findings

OCT findings comparing culprit PE to non-culprit plaques have been shown in Table 2. Culprit PE had smaller MLA and MFA and were longer than non-culprit plaques. Compared with non-culprit plaques, there were more TCFAs (26.0% vs. 11.9%, *p* <0.001) in culprit PE. Although LRPs were observed in both groups (53.5% of culprit PE and 37.9% of non-culprit plaques), LRPs underlying culprit PE had a larger mean and maximum lipid arc as well as a larger lipid index and more TCFAs compared to non-culprit plaques. Other underlying plaque characteristics—including cholesterol crystals, macrophages and microchannels—were comparable between the two groups. While there was no difference in frequency of calcification or spotty calcium comparing culprit PE to non-culprit plaques, when present, calcium appeared to be more extensive in culprit PE than in non-culprit plaques.

### Plaque Distribution

**Figure 1:**
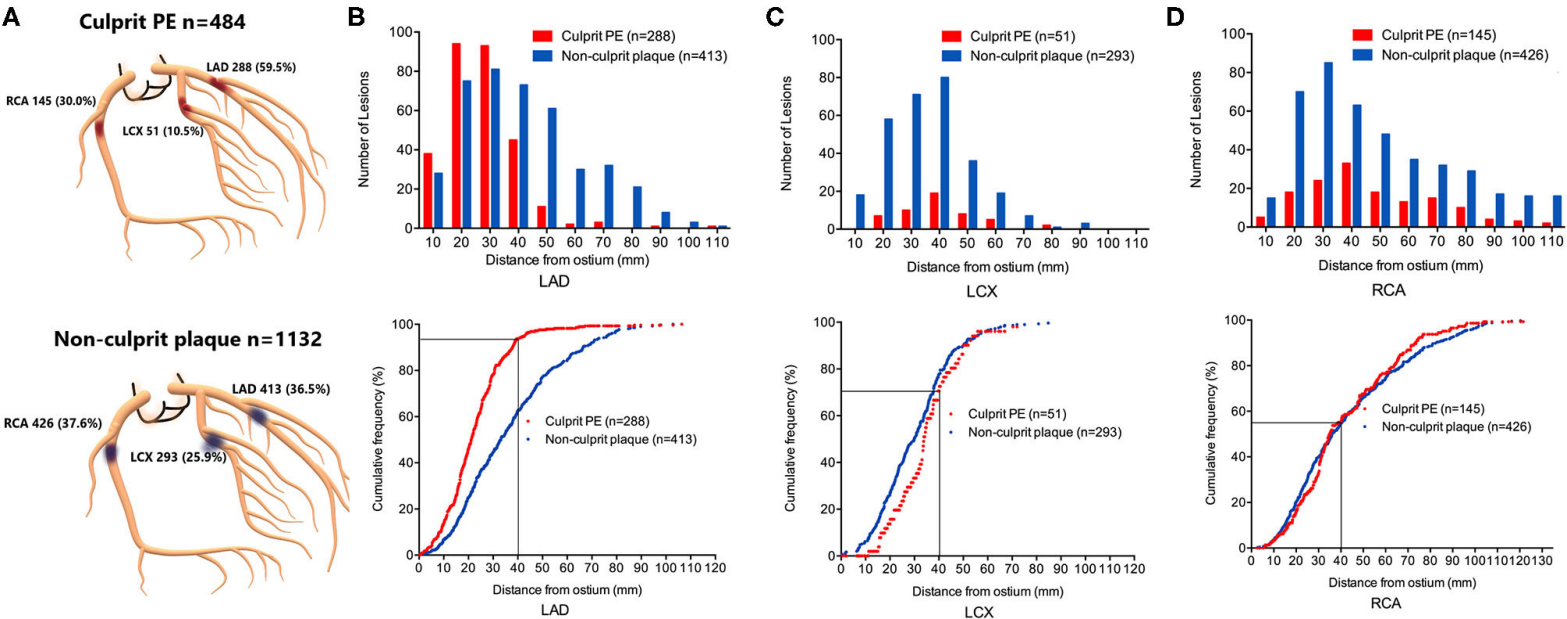
Location and longitudinal distribution of culprit PE and non-culprit plaques in STEMI patients with culprit plaque erosion. **(A)** Culprit PE in STEMI patients were mainly in the LAD, followed by RCA and LCX. Non-culprit plaques were evenly distributed in the RCA and LAD, followed by the LCX. The number of lesions and cumulative frequency of culprit PE and non-culprit plaques every 10 mm axial distance from the ostium of the LAD **(B)**, LCX **(C)**, and RCA **(D)** were shown. 93.8% of culprit eroded plaques in the LAD were within the proximal 40 mm of the artery. Among all plaques (culprit and non-culprit) within the proximal 40 mm of the LAD, over half represented culprit plaque erosions that were responsible for the acute event. LAD, left anterior descending artery; LCX, left circumflex artery; PE, plaque erosion; RCA, right coronary artery.

As shown in Figure 1, culprit PE was preferentially located in the LAD (288 of 484, 59.5%), followed by the RCA (145 of 484, 30.0%); they were least common in the LCX (51 of 484, 10.5%). Non-culprit plaques in the 484 patients with culprit PE were relatively evenly distributed in the RCA (426 of 1,132, 37.6%), LAD (413 of 1,132, 36.5%), and LCX (293 of 1,132, 25.9%) (Figure 1A).

Longitudinal mapping of both culprit PE and non-culprit plaques has been shown in Figures 1B–D. There was a gradient in the absolute number of culprit PE and non-culprit plaques from proximal to distal coronary segments mainly in the LAD and LCX while they were more evenly distributed in the RCA. Especially in the LAD, there was a strong proximal clustering of culprit PE compared with non-culprit plaques. Among all 527 culprit and non-culprit plaques within 40 mm of the LAD ostium, more than half (270 of 527, 51.2%) were a culprit PE that was responsible for the acute event.

The cumulative frequency distribution curves demonstrated that 93.8% (270 of 288) of culprit PE in the LAD were within 40 mm of the LAD ostium (Figure 1B), 70.6% of culprit PE in the LCX were within 40 mm of the LCX ostium (Figure 1C), and 55.2% of culprit PE in the RCA were within 40 mm of the RCA ostium (Figure 1D).

**Figure 2:**
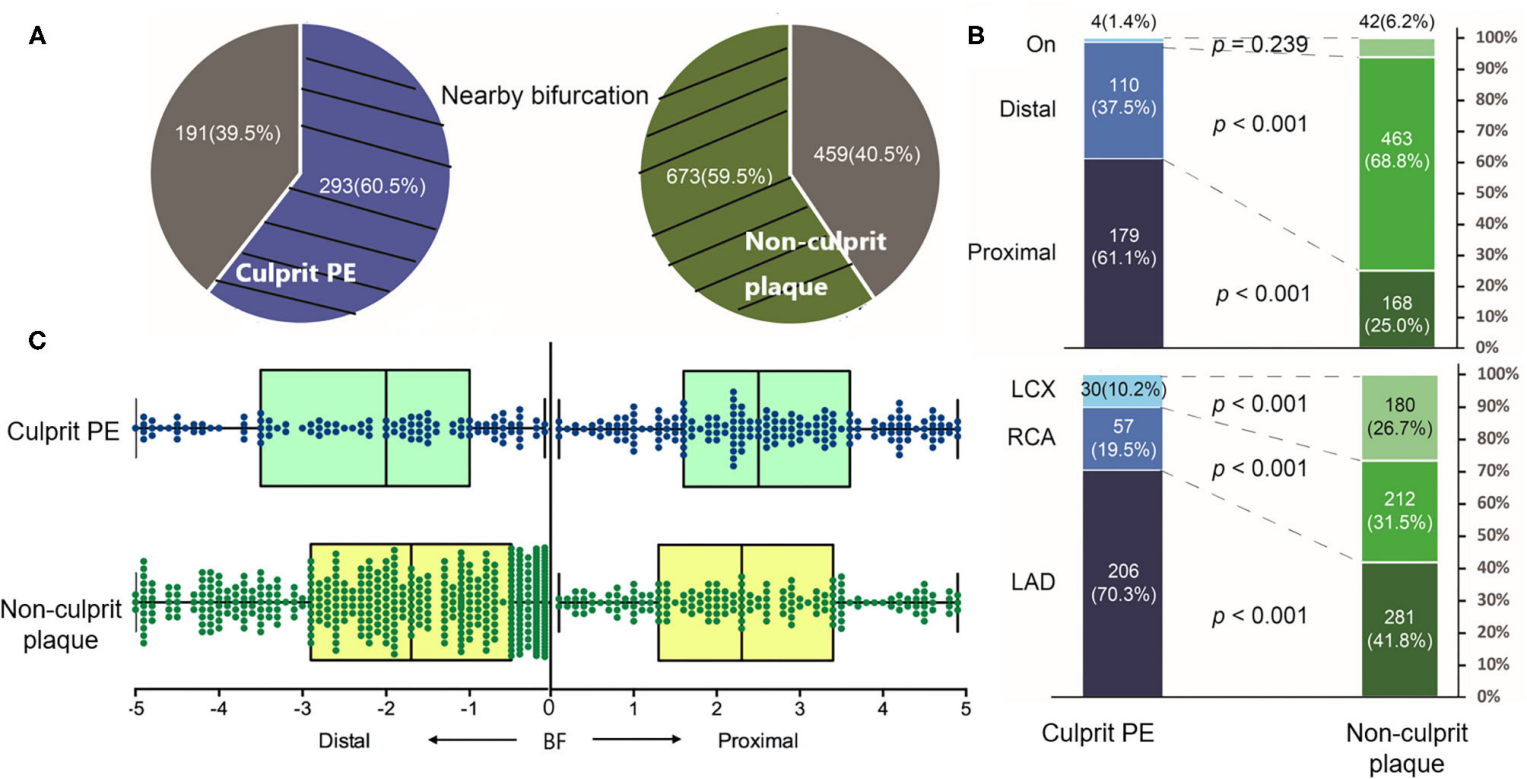
The distribution of culprit PE and non-culprit plaques near a bifurcation. **(A)** 60.5% culprit PE and 59.5% non-culprit plaques were located near a bifurcation. **(B)** Among them, culprit PE were mainly located in the LAD (70.3%) and most of them (61.1%) clustered proximal to a nearby bifurcation. On the contrary, most non-culprit plaques (68.8%) clustered distal to a nearby bifurcation. **(C)** Distance from culprit PE or the non-culprit plaque to the nearby bifurcation was shown. LAD, left anterior descending artery; LCX, left circumflex artery; PE, plaque erosion; RCA, right coronary artery.

Overall, 60.5% (293 of 484) culprit PE and 59.5% (673 of 1,132) non-culprit plaques were located near a bifurcation (Figure 2A). Among them, culprit PE showed a significant tendency to cluster proximal to a nearby bifurcation (proximal vs. distal: 61.1% vs. 37.5%, *p* <0.001) while the trend was completely opposite in non-culprit plaques (proximal vs. distal: 25.0% vs. 68.8%, *p* <0.001) (Figure 2B). This was especially true in the LAD where 70.3% of culprit PE were near a bifurcation, but it was less common in the RCA (19.5%) or LCX (10.2%). In contrast, non-culprit plaques were near a bifurcation in 41.8% of LAD, 31.5% of RCA, and 26.7% of LCX (Figure 2B). Culprit PE that were located proximally were within 2.5 (1.6–3.6) mm from the nearby bifurcation while non-culprit PE that were located distally were within 1.7 (0.5–2.9) (Figure 2C).

### Predictors of Culprit PE (vs. Non-culprit Plaques)

**Figure 3:**
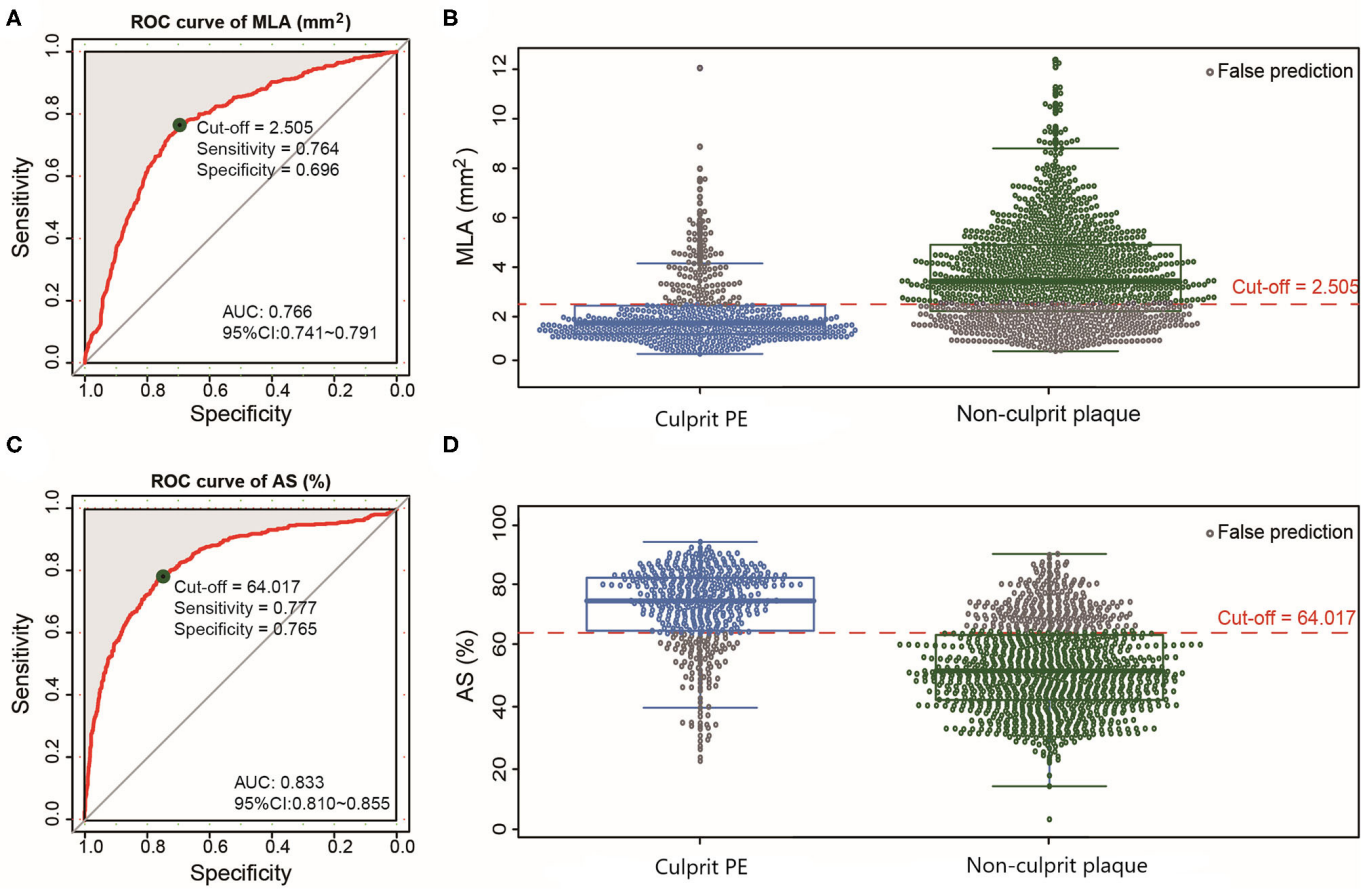
ROC curves of MLA and AS for differentiating culprit PE from non-culprit plaques. The optimal cut-off value of MLA was 2.51 mm^2^ (AUC = 0.766) **(A)** and the prediction probability was 76.4% for culprit PE **(B)**. The optimal cut-off value of AS was 64.02% (AUC = 0.833) **(C)** and the prediction probability was 77.7% for culprit PE **(D)**. AS, area stenosis; AUC, area under the curve; CI, confidence interval; MLA, minimal lumen area; PE, plaque erosion; ROC, receiver operating characteristic.

The inferred pre-thrombotic MLA was smaller and AS was more severe in culprit PE than in non-culprit plaques (Table 2). According to the ROC analysis, the optimal cut-off values of MLA <2.51 mm^2^ [area under the curve (AUC) = 0.766] and AS >64.02% (AUC = 0.833) could distinguish culprit PE from non-culprit plaques with a sensitivity of 76.4 and 77.7%, a specificity of 69.6 and 76.5%, a positive predictive value of 51.8 and 58.6%, a negative predictive value of 87.4 and 88.9%, and a diagnostic accuracy of 71.7 and 76.9% (Figures 3A–D).

**Figure 4:**
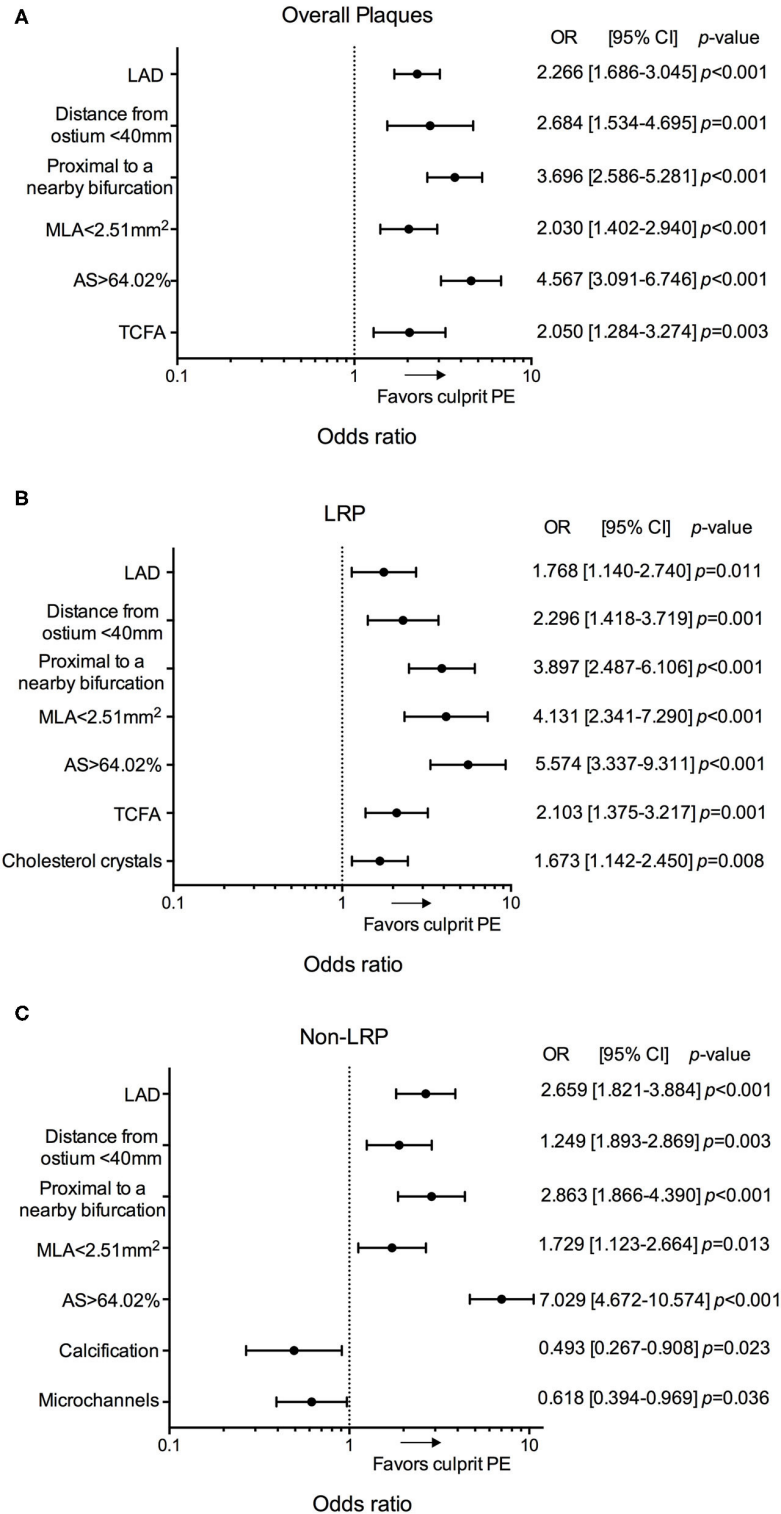
Predictors for culprit PE in overall cohort of 484 patients. Odds ratios for the presence of culprit PE overall **(A)**, in LRPs **(B)**, and in non-LRPs **(C)** according to LAD location, distance from the coronary ostium <40 mm, proximal to a nearby bifurcation, MLA <2.51 mm^2^, AS > 64.02%, TCFA, presence of cholesterol crystals, calcification and microchannels. AS, area stenosis; CI, confidence interval; LAD, left anterior descending artery; LRP, lipid-rich plaque; MLA, minimal lumen area; OR, odds ratio; PE, plaque erosion; TCFA, thin-cap fibroatheroma.

In the multivariable analysis, location in the LAD, distance from ostium <40 mm, proximal to a nearby bifurcation, MLA <2.51 mm^2^, AS > 64.02%, and presence of TCFA were significantly associated with culprit PE (Figure 4, Supplementary Table 2). Subgroup analyses of different underlying phenotypes showed that location in the LAD, distance from ostium <40 mm, proximal to a nearby bifurcation, MLA <2.51 mm^2^, and AS > 64.02% were predictive of culprit PE, regardless of underlying plaque phenotype (LRPs or non-LRPs). TCFA and presence of cholesterol crystals were significantly associated with culprit PE in the subgroup with underlying LRP morphology while less calcification and microchannels were significantly associated with culprit PE in the subgroup of non-LRPs (Figure 4, Supplementary Tables 3, 4).

**Figure 5:**
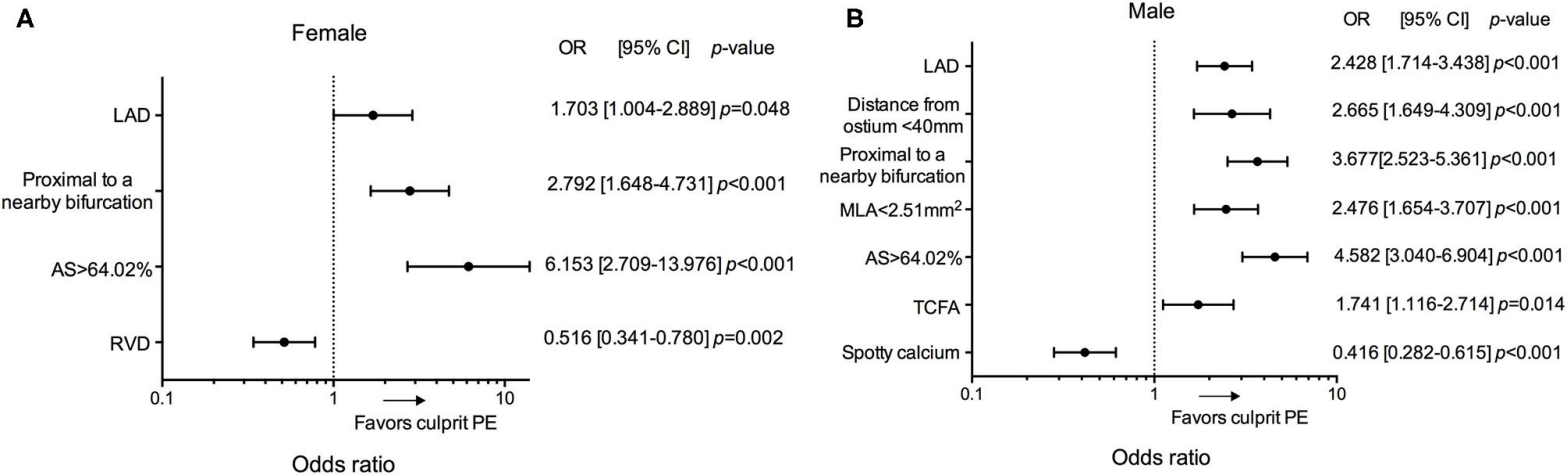
Subgroup analyses of predictors for culprit PE in patients according to sex. Odd ratios for the presence of culprit PE in female **(A)** and male **(B)** according to LAD location, distance from the coronary ostium <40 mm, proximal to a nearby bifurcation, MLA <2.51 mm^2^, AS >64.02%, RVD, TCFA and spotty calcium. AS, area stenosis; CI, confidence interval; LAD, left anterior descending artery; MLA, minimal lumen area; OR, odds ratio; PE, plaque erosion; RVD, reference vessel diameter; TCFA, thin-cap fibroatheroma.

In an exploratory analysis the predictors of culprit PE in male and female were then investigated (Figure 5, Supplementary Tables 5, 6). Location in the LAD, proximal to a nearby bifurcation, and AS > 64.02% were common predictors for culprit PE, regardless of sex. Distance from coronary ostium <40 mm, MLA <2.51 mm^2^, TCFA, and less spotty calcium were risk factors of culprit PE in males, but not in females. Smaller RVD was associated with culprit PE only in females. No co-linearity was found between MLA and AS, MLA <2.51 mm^2^ and AS>64.02% in overall plaques and subgroup of LRPs vs. non-LRPs and males vs. females.

## Discussion

This is the first study investigating characteristics and predictors of erosion-prone plaques in a comprehensive map of culprit and non-culprit sites in STEMI patients in whom the acute event was caused by culprit PE. The main findings were as follows. (1) Culprit PE were highly populated at “hot spots” within the proximal 40 mm of the LAD. (2) Culprit PE tended to develop proximal to a nearby bifurcation, especially in the LAD. (3) MLA <2.51 mm^2^ and AS >64.02% were the optimal cut-off values of luminal stenosis to discriminate culprit eroded plaques from non-eroded, non-culprit plaques. (4) Similarities and differences in predictors of culprit PE were found between different plaque phenotypes and between males and females.

### Location and Spatial Distribution of Plaque Erosion

Like other high-risk plaques (ruptures and TCFAs) (14–16) and acute coronary occlusions (9), we found non-uniform PE distribution with proximal clustering (within 40 mm of the coronary ostium) in the LAD (93.8%) and LCX (70.6%). Notably, the present study confirmed and extended the results of our prior OCT studies (2, 4), verifying the presence of specific “hot spots” for culprit PE (within the proximal 40 mm of the LAD) where culprit PE accounted for more than half of all (culprit and non-culprit) plaques. Moreover, LAD location and distance from coronary ostium <40 mm were strong predictors of culprit PE.

The etiology for the LAD predominance and proximal clustering still remains unclear. Although the LAD and LCX taper more than the RCA, vessel size seems not to be a good explanation for proximal clustering because RVD was not significantly associated with culprit PE. Compared with other arterial segments, the proximal LAD has multiple sidebranches leading to marked variations in blood instability and shear stress. Furthermore, there is lower endothelial shear stress (ESS) in the proximal compared to the distal segment of the left coronary artery (17).

### Nearby Bifurcation and Plaque Erosion

In the present study culprit PE tended to cluster proximal to a nearby bifurcation, a sharply distinct distribution pattern compared with non-culprit plaques. The magnitude of wall shear stress (WSS) and OSI around a bifurcation is very variable as proved using computational fluid dynamics simulations (18). Disturbed flow and oscillatory WSS can influence the site of atherosclerotic plaque formation and development of culprit PE. Low ESS has been associated with lipid, and high ESS has been correlated with the site of acute erosion and thrombosis (19). Moreover, reduced ability to repair wounds of endothelial cells near sidebranches compared with non-branch regions might also facilitate culprit PE formation (20).

### Luminal Narrowing and Plaque Erosion

Although OCT imaging cannot evaluate the extent of plaque burden due to its limited imaging depth, culprit PE presented with a higher degree of pre-existing luminal narrowing compared with non-culprit plaques. Although we do not really know the precise culprit PE luminal narrowing before thrombus formation, MLA and AS (excluding thrombus) were measured in the current study in order to approach the pre-thrombus culprit luminal narrowing as much as possible. The best cut-off values of MLA (<2.51 mm^2^) and AS (>64.02%) were found to be common and strong predictors of culprit PE, regardless of the underlying substrates. These results suggested that pre-existing severe lumen narrowing was still important for an erosion-prone plaque to turn into a culprit PE at geometrically predisposed areas. Whether erosion-prone plaques experience a rapid step-wise progression (21) at the onset of the acute coronary event remains unclear. Meanwhile, distinct level of ESS, ESS gradient (ESSG), and OSI have been found in upstream and downstream to the MLA at the location of eroded plaques and thrombus (22); and the degree of luminal narrowing could further influence composition of thrombus (23) and healing in eroded plaques (24). Future prospective imaging studies and computational fluid dynamics studies are needed to establish association among dynamic luminal narrowing, shear stress, and clinical events caused by culprit erosion.

### Predictors of Culprit PE

Besides luminal narrowing and focal geometry, the presence of TCFA remained one risk factor for culprit PE overall and in the subgroup with underlying LRP. Different from TCFA as a precursor for plaque rupture, TCFA in PE might merely represent greater lipid accumulation and not be pathophysiologically linked; there was more lipid, but comparable thinnest FCT. As a heterogeneous entity, underlying LRPs accounted for about half of culprit PE (2). Notably, cholesterol crystals were found to be independent predictors of culprit PE in the subgroup of LRPs. Cholesterol crystals are mainly taken up by macrophages and exert effects on different cell types such as neutrophils and endothelial and smooth muscle cells (25). Through stimulating neutrophil extracellular traps releasing and activation of the complement system, cholesterol crystals could facilitate thrombosis (25). Because the role of cholesterol crystals has been mainly elucidated in plaque rupture, the exact physiological mechanisms of cholesterol crystals in PE remains unknown. Nevertheless, our results suggested that erosion-prone vulnerable patients might still benefit from statin therapy.

In the subgroup of non-LRPs (fibrous and fibrocalcific plaques), the absence of calcification and microchannels was a risk factor for culprit PE. The pattern and extent of calcification tended to differ sharply according to plaque phenotype (26). In line with our findings, previous pathological studies have reported less histological calcification in erosions and maximum calcification in fibrocalcific plaques compared with other phenotypes (26). On one hand, calcification has been strongly associated with adverse outcomes and represents increased risk of coronary artery disease (26, 27). On the other hand, in an erosion-prone vulnerable patient, calcification seemed to represent a lower risk for a plaque turning into a culprit PE. Microchannels or neoangiogenesis at non-culprit regions has been identified as potential predictors of angiographic plaque progression and multiple plaque ruptures through leak of inflammatory cells and cytokines (12, 28). However, pathological and imaging data about the association of microchannels with plaque erosion are still limited.

### Sex Difference in Predictors of Culprit PE

Less non-culprit rupture and calcium content has been observed in females (29). Unlike in males, plaque phenotype, lipid content, and micro-structures did not appear to be important for culprit PE in females while location and plaque burden were key predictors of culprit PE. Interestingly, smaller RVD was found to be a risk factor for culprit PE in females, but not in males. Females have significantly smaller epicardial coronary arteries together with higher baseline myocardial blood flow compared with males, resulting in an increase in ESS (29). In this case, vessel segments with smaller RVD might be more sensitive to disturbed flow and rapid progression of plaque burden in females. Unlike rupture-prone vulnerability, our study found a negative role of spotty calcium in culprit PE in males.

### Study Limitations

First, this was a retrospective observational analysis although data were collected prospectively. Second, consecutive patients undergoing OCT imaging of one, two, or three major epicardial coronary arteries were enrolled in order to minimize selection bias; and non-culprit plaques were analyzed in all of the imaged vessels. Had we included only patients with 3-vessel OCT, there would have been a different bias. Third, as the current OCT system cannot visualize individual endothelial cells, the OCT definition of culprit PE was in some ways an exclusion diagnosis. Fourth, the features of the culprit plaques were analyzed just after deocclusion; and correct underlying plaque analysis might be impeached due to the signal absorption caused by remnant thrombus. In the current study, the proportion of red thrombus was comparable between the two groups; and patients with massive thrombus in the culprit lesion were excluded. Fifth, manual thrombectomy could also alter the morphology of the culprit lesion by inducing dissection or iatrogenic rupture. Sixth, the data reflected the focal geometry and characteristics of erosion-prone coronary plaques of Chinese STEMI patients. Thus, the results may not be generalizable to other countries or ethnicities or to NSTE-ACS patients. Seventh, to date, there is no diagnostic definition for a plaque that is going to develop culprit PE and occlusive thrombus. Through providing comprehensive information of culprit PE and non-culprit lesions in other locations besides the culprit site, the present study only elucidated focal geometry and characteristics of erosion-prone coronary plaques in erosion-prone vulnerable patients. Finally, while not currently available, long-term follow-up of this large series of STEMI patients is in progress.

## Conclusions

**Figure 6:**
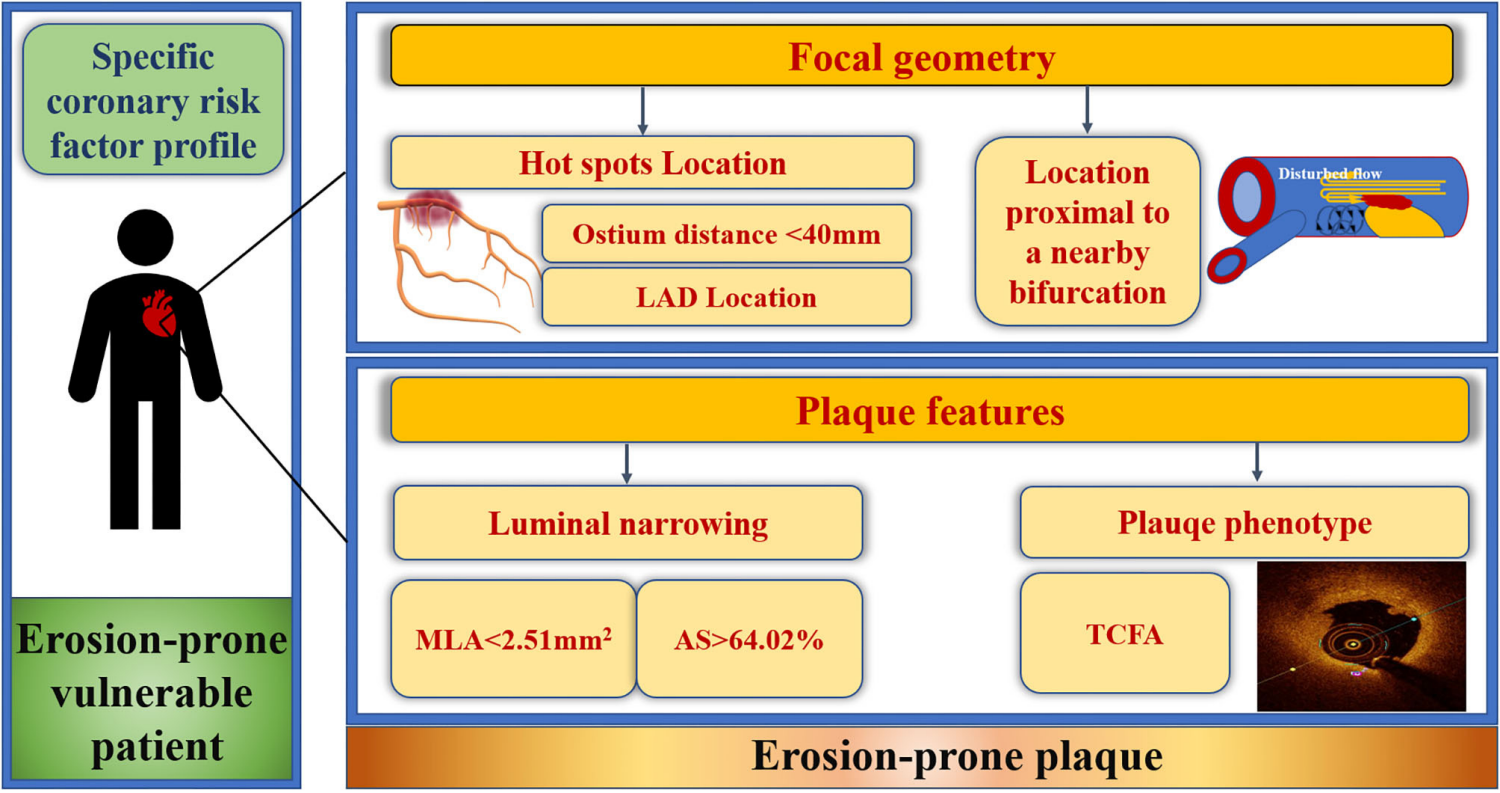
Focal geometry and characteristics of erosion-prone plaques. Culprit plaque erosions selectively developed at predisposed areas within the coronary artery tree and exhibited specific features with certain focal geometry that can be used for risk-stratifying high-risk erosion-prone plaques in erosion-prone vulnerable STEMI patients. Culprit plaque erosion tended to cluster within the “hot spots,” defined as distance from the ostium to the MLA <40 mm in the LAD. LAD location, distance from coronary ostium <40 mm, proximal to a nearby bifurcation, MLA <2.51 mm^2^, AS > 64.02% and underlying TCFA of the culprit lesion were predictors of culprit plaque erosion. AS, area stenosis; LAD, left anterior descending artery; MLA, minimal lumen area; STEMI, ST-segment elevation myocardial infarction; TCFA, thin-cap fibroatheroma.

The current study elucidated particular focal geometry (anatomy and location) and characteristics for erosion-prone plaques. LAD location, distance from the coronary ostium <40 mm, location proximal to a nearby bifurcation, MLA <2.51 mm^2^, AS >64.02%, and presence of TCFA were independent predictors for culprit PE overall, which can be used for risk-stratifying high-risk erosion-prone plaques in erosion-prone vulnerable patients (Figure 6). Cholesterol crystals were predictive of culprit PE with underlying LRP morphology while the absence of calcification and microchannels were risk factors for culprit PE with an underlying non-LRP. Specific risk factors of erosion-prone plaques should be considered in men and women. Future prospective *in vivo* studies are required to validate the predictive value for clinical events or efficient therapeutic targets of plaque erosion.

## Data Availability Statement

The raw data supporting the conclusions of this article will be made available by the authors, without undue reservation.

## Ethics Statement

The studies involving human participants were reviewed and approved by The Ethics Committee of the 2nd Affiliated Hospital of Harbin Medical University (Harbin, China). The patients/participants provided their written informed consent to participate in this study.

## Author Contributions

MC: conception and design of the research, acquisition, analysis and interpretation of data, manuscript drafting, and critical manuscript revision. TW, JZ, and ZD: substantial contribution to data acquisition and analysis. ZW and LL: contribution to statistical analysis. GW: substantial contribution to patients' enrollment and cardiac intervention. JT and HJ: critical manuscript revision. GM and BY: substantial contribution to the design of research and critical manuscript revision. All authors contributed to the article and approved the submitted version.

## Funding

This work was supported by National Key R&D Program of China (grant No. 2016YFC1301100 to BY) and National Natural Science Foundation of China (grant No. 81827806).

## Conflict of Interest

GM has received research and fellowship support grants from Abbott; has been a consultant for and has received honoraria from Boston Scientific and Phillips; and has been a consultant for Terumo. The remaining authors declare that the research was conducted in the absence of any commercial or financial relationships that could be construed as a potential conflict of interest.

## Publisher's Note

All claims expressed in this article are solely those of the authors and do not necessarily represent those of their affiliated organizations, or those of the publisher, the editors and the reviewers. Any product that may be evaluated in this article, or claim that may be made by its manufacturer, is not guaranteed or endorsed by the publisher.

## References

[1] Libby P Pasterkamp G Crea F Jang IK. Reassessing the mechanisms of acute coronary syndromes. Circ Res. (2019) 124:150–60. 10.1161/CIRCRESAHA.118.311098 30605419 PMC6447371

[2] Cao M Zhao L Ren X Wu T Yang G Du Z. Pancoronary plaque characteristics in STEMI caused by culprit plaque erosion versus rupture: 3-vessel OCT study. JACC Cardiovasc Imaging. (2021) 14:1235–45. 10.1016/j.jcmg.2020.07.047 33129735

[3] Crea F Libby P. Acute coronary syndromes: the way forward from mechanisms to precision treatment. Circulation. (2017) 136:1155–66. 10.1161/CIRCULATIONAHA.117.029870 28923905 PMC5679086

[4] Dai J Xing L Jia H Zhu Y Zhang S Hu S. *In vivo* predictors of plaque erosion in patients with ST-segment elevation myocardial infarction: a clinical, angiographical, and intravascular optical coherence tomography study. Eur Heart J. (2018) 39:2077–85. 10.1093/eurheartj/ehy101 29547992

[5] Sato A. Plaque erosion is a predictable clinical entity and tailored management in patients with ST-segment elevation myocardial infarction. J Thorac Dis. (2018) 10:S3274–5. 10.21037/jtd.2018.08.103 30370135 PMC6186580

[6] Yamamoto E Yonetsu T Kakuta T Soeda T Saito Y Yan BP. Clinical and laboratory predictors for plaque erosion in patients with acute coronary syndromes. J Am Heart Assoc. (2019) 8:e012322. 10.1161/JAHA.119.012322 31640466 PMC6898801

[7] Jinnouchi H Virmani R Finn AV. Are characteristics of plaque erosion defined by optical coherence tomography similar to true erosion in pathology? Eur Heart J. (2018) 39:2086–9. 10.1093/eurheartj/ehy113 29547936

[8] Jia H Abtahian F Aguirre AD Lee S Chia S Lowe H. *In vivo* diagnosis of plaque erosion and calcified nodule in patients with acute coronary syndrome by intravascular optical coherence tomography. J Am Coll Cardiol. (2013) 62:1748–58. 10.1016/j.jacc.2013.05.071 23810884 PMC3874870

[9] Wang JC Normand SL Mauri L Kuntz RE. Coronary artery spatial distribution of acute myocardial infarction occlusions. Circulation. (2004) 110:278–84. 10.1161/01.CIR.0000135468.67850.F4 15249505

[10] Dai J Fang C Zhang S Li L Wang Y Xing L. Frequency, predictors, distribution, and morphological characteristics of layered culprit and nonculprit plaques of patients with acute myocardial infarction: *in vivo* 3-vessel optical coherence tomography study. Circ Cardiovasc Interv. (2020) 13:e009125. 10.1161/CIRCINTERVENTIONS.120.009125 32957793

[11] Sugiyama T Yamamoto E Bryniarski K Xing L Lee H Isobe M. Nonculprit plaque characteristics in patients with acute coronary syndrome caused by plaque erosion vs plaque rupture: a 3-vessel optical coherence tomography study. JAMA Cardiol. (2018) 3:207–14. 10.1001/jamacardio.2017.5234 29417141 PMC5885886

[12] Vergallo R Uemura S Soeda T Minami Y Cho JM Ong DS. Prevalence and predictors of multiple coronary plaque ruptures: *in vivo* 3-vessel optical coherence tomography imaging study. Arterioscler Thromb Vasc Biol. (2016) 36:2229–38. 10.1161/ATVBAHA.116.307891 27634834

[13] Amabile N Hammas S Fradi S Souteyrand G Veugeois A Belle L. Intra-coronary thrombus evolution during acute coronary syndrome: regression assessment by serial optical coherence tomography analyses. Eur Heart J Cardiovasc Imaging. (2015) 16:433–40. 10.1093/ehjci/jeu228 25428947

[14] Cheruvu PK Finn AV Gardner C Caplan J Goldstein J Stone GW. Frequency and distribution of thin-cap fibroatheroma and ruptured plaques in human coronary arteries: a pathologic study. J Am Coll Cardiol. (2007) 50:940–9. 10.1016/j.jacc.2007.04.086 17765120

[15] Wykrzykowska JJ Mintz GS Garcia-Garcia HM Maehara A Fahy M Xu K. Longitudinal distribution of plaque burden and necrotic core-rich plaques in nonculprit lesions of patients presenting with acute coronary syndromes. JACC Cardiovascu Imaging. (2012) 5:S10–8. 10.1016/j.jcmg.2012.01.006 22421223

[16] Araki M Soeda T Kim HO Thondapu V Russo M Kurihara O. Spatial distribution of vulnerable plaques: comprehensive *in vivo* coronary plaque mapping. JACC Cardiovasc Imaging. (2020) 13:1989–99. 10.1016/j.jcmg.2020.01.013 32912472

[17] Soulis JV Farmakis TM Giannoglou GD Louridas GE. Wall shear stress in normal left coronary artery tree. J Biomech. (2006) 39:742–9. 10.1016/j.jbiomech.2004.12.026 16439244

[18] Lee SW Antiga L Spence JD Steinman DA. Geometry of the carotid bifurcation predicts its exposure to disturbed flow. Stroke. (2008) 39:2341–47. 10.1161/STROKEAHA.107.510644 18556585

[19] Thondapu V Mamon C Poon EKW Kurihara O Kim HO Russo M. High spatial endothelial shear stress gradient independently predicts site of acute coronary plaque rupture and erosion. Cardiovasc Res. (2020) 117:1974–85. 10.1093/cvr/cvaa251 32832991

[20] Akong TA Gotlieb AI. Reduced *in vitro* repair in endothelial cells harvested from the intercostal ostia of porcine thoracic aorta. Arterioscler Thromb Vasc Biol. (1999) 19:665–671. 10.1161/01.atv.19.3.665 10073971

[21] Jang IK. Plaque progression: slow linear or rapid stepwise? Circ Cardiovasc Imaging. (2017) 10:e006964. 10.1161/CIRCIMAGING.117.006964 28893799

[22] Yamamoto E Thondapu V Poon E Sugiyama T Fracassi F Dijkstra J. Endothelial shear stress and plaque erosion: a computational fluid dynamics and optical coherence tomography study. JACC Cardiovasc Imaging. (2019) 12:374–5. 10.1016/j.jcmg.2018.07.024 30343069

[23] Kurihara O Takano M Soeda T Fracassi F Araki M Nakajima A. Degree of luminal narrowing and composition of thrombus in plaque erosion. J Thromb Thrombol. (2020) 1:143–50. 10.1007/s11239-020-02159-8 32472306

[24] Vergallo R Crea F. Atherosclerotic plaque healing. N Engl J Med. (2020) 383:846–57. 10.1056/NEJMra2000317 32846063

[25] Baumer Y Mehta NN Dey AK Powell-Wiley TM Boisvert WA. Cholesterol crystals and atherosclerosis. Eur Heart J. (2020) 41:2236–39. 10.1093/eurheartj/ehaa505 32572475 PMC7850045

[26] Mori H Torii S Kutyna M Sakamoto A Finn AV Virmani R. Coronary artery calcification and its progression: what does it really mean? JACC Cardiovasc Imaging. (2018) 11:127–42. 10.1016/j.jcmg.2017.10.012 29301708

[27] Puchner SB Mayrhofer T Park J Lu MT. Differences in the association of total versus local coronary artery calcium with acute coronary syndrome and culprit lesions in patients with acute chest pain: the coronary calcium paradox. Atherosclerosis. (2018) 274:251–7. 10.1016/j.atherosclerosis.2018.04.017 29703635 PMC5999579

[28] Uemura S Ishigami K Soeda T Okayama S Sung JH Nakagawa H. Thin-cap fibroatheroma and microchannel findings in optical coherence tomography correlate with subsequent progression of coronary atheromatous plaques. Eur Heart J. (2012) 33:78–85. 10.1093/eurheartj/ehr284 21831910

[29] Haider A Bengs S Luu J Osto E Siller-Matula JM Muka T. Sex and gender in cardiovascular medicine: presentation and outcomes of acute coronary syndrome. Eur Heart J. (2020) 41:1328–36. 10.1093/eurheartj/ehz898 31876924

