# Supplementary material for: Focal Geometry and Characteristics of Erosion-Prone Coronary Plaques in vivo Angiography and Optical Coherence Tomography Study
Source: Front Cardiovasc Med. 2021 Sep 8;8:709480. doi: 10.3389/fcvm.2021.709480 (PMC8457312; doi:10.3389/fcvm.2021.709480)
Supplement: Supplementary file 1 [file Data_Sheet_1.docx]

**Supplementary Methods**

**Definition and diagnosis of coronary risk factors**

Patients with active smoking within 1 month and no smoking for >1 month were defined as current smokers and former smokers, respectively. Diabetes mellitus was diagnosed if a patient met 1 of the following criteria: documented history or self-reported clinician diabetes mellitus, taking hypoglycemic medicine, fasting glucose ≥126mg/dL, 2h plasma glucose level ≥200mg/dL, classic symptom with casual plasma glucose level ≥200mg/dL, or hemoglobin A1c (HbA1c) ≥6.5%. Hypertension was diagnosed as systolic blood pressure ≥140mmHg or diastolic blood pressure ≥90mmHg or current use of anti-hypertensive agents. Dyslipidemia was defined as total cholesterol (TC) level ≥220mg/dL, triglycerides ≥150 mg/dL, low-density lipoprotein cholesterol (LDL-C) ≥140mg/dL, high-density lipoprotein cholesterol (HDL-C) ≤40mg/dL, or current use of agents for dyslipidemia(1). Estimated glomerular filtration (eGFR) was calculated according to the 2009 Chronic Kidney Disease Epidemiology Collaboration (CKD-EPI) equation. Chronic kidney disease (CKD) was diagnosed as eGFR <60mL/min per 1.73m^2^(2).

**Quantitative and qualitative OCT analyses of underlying plaques**

Lipid was a signal-poor region with a poorly defined or diffuse border; and the lipid arc and length were measured, and a lipid index was derived as the product of mean lipid arc x lipid length. Fibrous cap thickness (FCT) was measured in lipidic plaques. Lipid-rich plaque (LRP) had a maximal lipid arc >90°. Thin-cap fibroatheroma (TCFA) was a plaque with maximal lipid arc >90° and thinnest FCT ≤65μm. Fibrous and fibrocalcific plaque were defined by a homogeneous OCT signal with high backscatter not meeting LRP definitions. Macrophages had highly backscattering focal regions within the fibrous cap. Microchannels were small (50-300μm), signal-poor structures with vesicular or tubular shape without connection to the lumen, recognized on ≥3 consecutive cross-sectional images. Cholesterol crystals were thin and linear regions of high signal intensity with high backscattering within a plaque. Calcification was defined as any signal-poor or heterogeneous area delimited by sharp borders. In particular, lesions subtending an arc ≤ 90° and extending in length less than 4mm were classified as spotty calcium. Calcification arcs were measured in each cross-sectional image, and lengths were obtained on the longitudinal view. Calcified Calcium index was derived as the product of mean calcium arc x calcium length. Thrombus was an irregular mass (diameter >250μm) adherent to the vessel wall or floating within the lumen(3). Thrombus was categorized as either erythrocyte-rich (red) thrombus, defined by high backscattering and high attenuation, or platelet-rich (white) thrombus, defined by homogeneous backscattering with low attenuation.

Proximal and distal references were the sites with the largest lumen area (LA) proximal and distal to the lesion, but within the same segment; and a mean reference LA was calculated. In image frames where luminal border was visible in at least three of four quadrants of the image, LA was traced using the “Area-Multiple Point” tool of the proprietary analysis software (St. Jude Medical) (Online Figure 2). When the luminal border was difficult to identify in more than one quadrant, LA was extrapolated from the nearest proximal or distal frame with visible lumen contour. Copy-paste function of the proprietary analysis software was used, supplemented by manual corrections to adjust the copied area to the visible parts of vessel lumen in the frame it was copied to. If needed, additional manual corrections were taken with the assistance of the longitudinal view(4,5).

**Supplementary figures**

STEMI patients with OCT

(n=2136)

STEMI patients suitable for both culprit and non-culprit lesion evaluation

(n=1931)

Pre-dilation (n=28)

In-stent restenosis or thrombosis (n=66)

Suboptimal image quality or short analyzable pullback or massive thrombus (n=81)

Incomplete demographic, clinical or imaging data (n=30)

Others:

Plaque ruptures (n=1219)

Calcified nodule (n=58)

Others (n=136)

Culprit lesion not identified (n=34)

Culprit plaque erosion

(n=484)

Non-culprit plaque

(n=1132)

STEMI patients caused by culprit plaque erosion

(n=484)

1-vessel OCT imaging (n=71)

2-vessel OCT imaging (n=114)

3-vessel OCT imaging (n=299)

1196 imaged vessels

**Supplementary Figure 1. Study flow chart.** Of 2136 STEMI patients with OCT imaging, 205 patients were excluded due to pre-dilation before OCT (n = 28), in-stent restenosis or thrombosis (n = 66), or suboptimal image quality or short analyzable pullback or massive thrombus (n = 81), incomplete demographic, clinical or imaging data (n = 30). Finally, a total of 484 STEMI patients caused by culprit plaque erosion were defined among 1931 patients in the study.

Abbreviations: OCT = optical coherence tomography; STEMI = ST-segment elevation myocardial infarction.

**
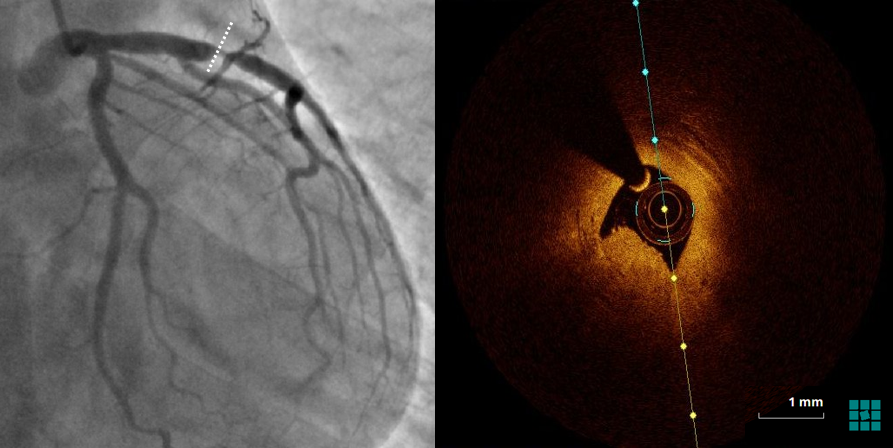
**

**Culprit plaque erosion**

**Patients with culprit plaque erosion**

**B**

**A**

**Left anterior descending artery**

**Supplementary Figure 2. Representative case of culprit plaque erosion.** (A) Coronary angiography showed a severe stenosis in the middle of the left anterior descending artery; (B) Plaque erosion with white thrombus (arrows).


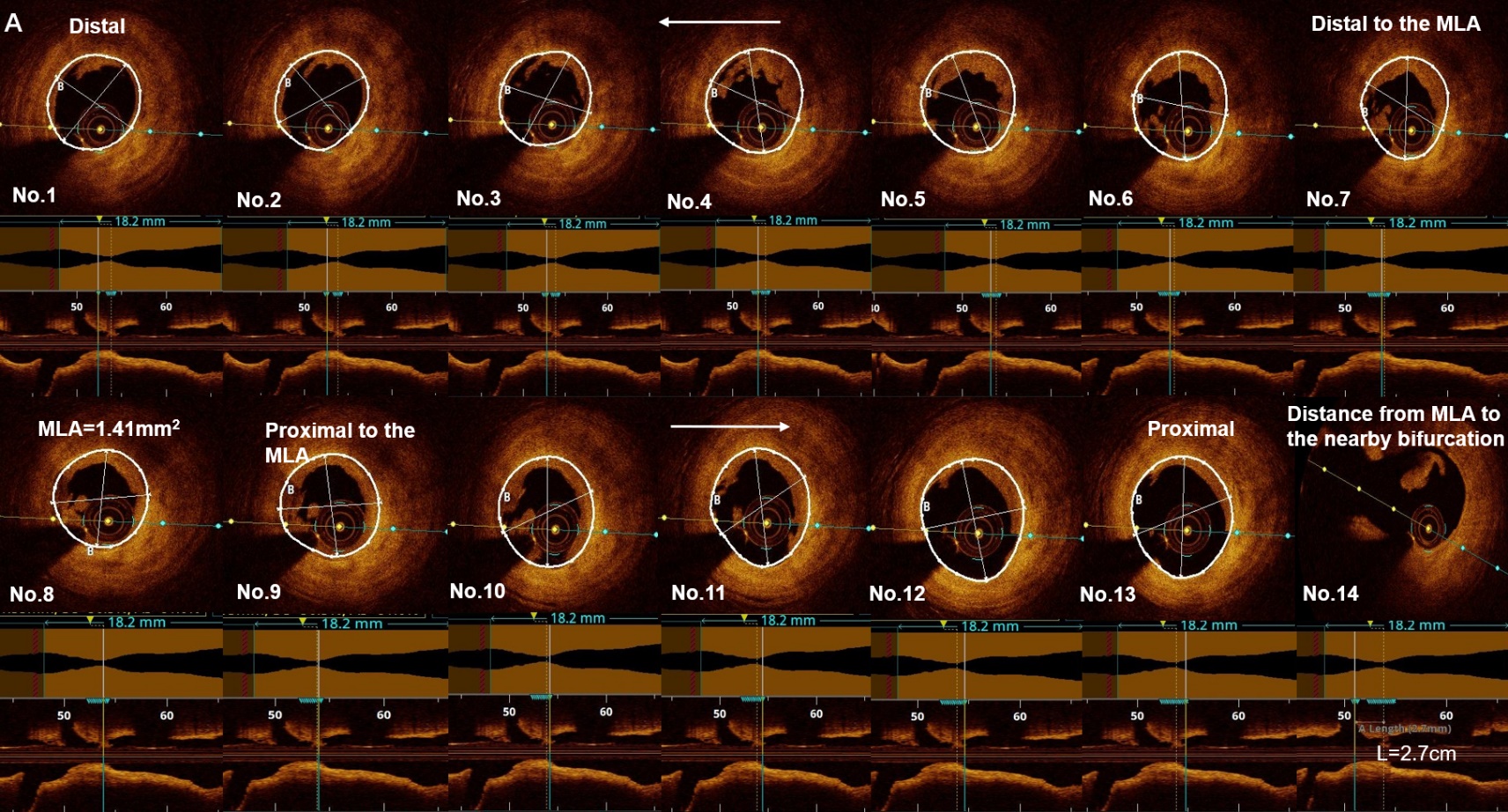

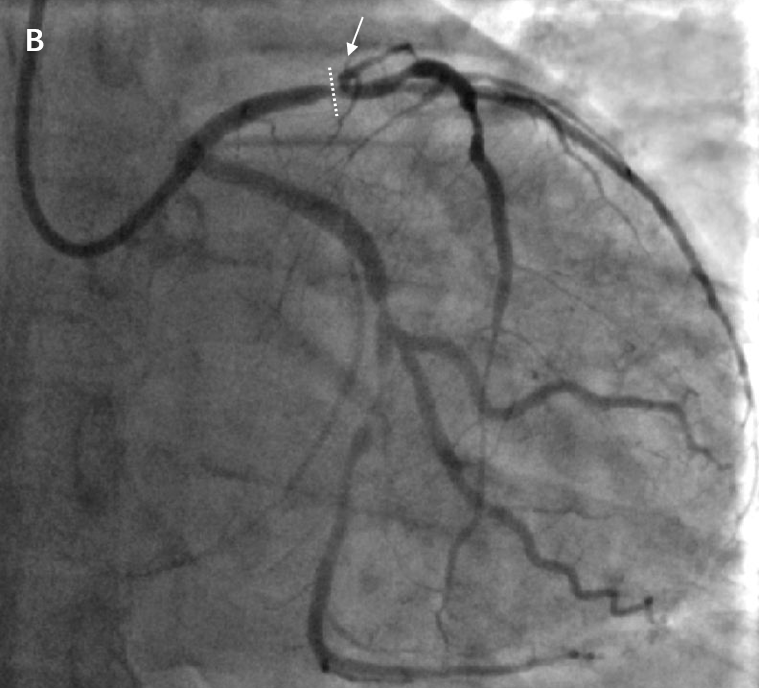


**Supplementary Figure 3. Representative measurement of distance from minimal lumen area (pre-thrombotic) to the nearby bifurcation in the culprit lesion.** (A) OCT images from No.13 to No.1 showed segment covered by thrombus in the culprit lesion from the proximal to the distal frame. LA was traced using the “Area-Multiple Point” tool of the proprietary analysis software (St. Jude Medical) in the proximal and distal to the MFA. MLA (1.41mm^2^) located in image No.8. MLA located in the proximal of the nearby bifurcation. Distance from MLA to the nearby bifurcation was 2.7cm (image No.14). (B) Coronary angiography showed a severe stenosis in the proximal of the left anterior descending artery, located in the proximal of the nearby bifurcation.

Abbreviations: LA = lumen area; MFA = minimal flow area; MLA = minimal lumen area; OCT = optical coherence tomography.

**Supplementary tables**

**Supplementary Table 1 Description of OCT pullbacks of the studied vessels**

| **Variables** | **LAD** | **RCA** | **LCX** | **Total** |
| --- | --- | --- | --- | --- |
| Total number of imaged vessels by OCT | 449/484 (92.8) | 393/484 (81.2) | 354/484 (73.1) | 1196/1452 (82.4) |
| OCT performed in the middle or distal segment | 396/449 (88.2) | 333/393 (84.7) | 228/354 (64.4) | 957/1196 (80.0) |
| Length of analyzed OCT pullbacks (mm) | 70.8 ± 25.5 | 84.1 ± 19.1 | 45.8 ± 15.5 | 206.3 ± 35.5 |

LAD = left anterior descending artery; LCX = left circumflex artery; OCT = optical coherence tomography; RCA = right coronary artery.

**Supplementary Table 2 Univariate and multivariate analysis of culprit PE in overall plaques**

| **Variables** | **Univariate analysis** | | | **Multivariate analysis** | | |
| --- | --- | --- | --- | --- | --- | --- |
|  | **OR** | **95%CI** | ***p*-value** | **OR** | **95%CI** | ***p*-value** |
| LAD | 3.251 | 2.271-4.654 | <0.001 | 2.266 | 1.686-3.045 | <0.001 |
| Distance from ostium <40mm | 3.301 | 1.986-5.486 | <0.001 | 2.684 | 1.534-4.695 | 0.001 |
| Proximal to a nearby bifurcation | 4.118 | 2.610-6.495 | <0.001 | 3.696 | 2.586-5.281 | <0.001 |
| MLA<2.51mm^2^ | 5.862 | 4.311-7.971 | <0.001 | 2.030 | 1.402-2.940 | <0.001 |
| AS>64.02% | 6.865 | 4.008-11.759 | <0.001 | 4.567 | 3.091-6.746 | <0.001 |
| RVD | 0.891 | 0.626-1.267 | 0.519 |  |  |  |
| LRP | 1.009 | 0.543-1.877 | 0.976 |  |  |  |
| TCFA | 2.819 | 1.619-4.908 | <0.001 | 2.050 | 1.284-3.274 | 0.003 |
| Cholesterol crystals | 1.630 | 0.989-2.688 | 0.055 | 1.048 | 0.748-1.467 | 0.786 |
| Macrophages | 1.449 | 0.903-2.324 | 0.124 |  |  |  |
| Calcification | 0.701 | 0.400-1.231 | 0.216 |  |  |  |
| Spotty calcium | 0.590 | 0.313-1.113 | 0.103 |  |  |  |
| Microchannels | 0.849 | 0.520-1.386 | 0.512 |  |  |  |

AS = area stenosis; CI = confidence interval; LAD = left anterior descending artery; LRP = lipid rich plaque; MLA = minimal lumen area; OR = odds ratio; PE = plaque erosion; RVD = reference vessel diameter; TCFA = thin-cap fibroatheroma.

**Supplementary Table 3 Univariate and multivariate analysis of culprit PE in LRPs**

| **Variables** | **Univariate analysis** | | | **Multivariate analysis** | | |
| --- | --- | --- | --- | --- | --- | --- |
|  | **OR** | **95%CI** | ***p*-value** | **OR** | **95%CI** | ***p*-value** |
| LAD | 3.485 | 2.167-5.604 | <0.001 | 1.768 | 1.140-2.742 | 0.011 |
| Distance from ostium <40mm | 5.202 | 2.921-9.263 | <0.001 | 2.296 | 1.418-3.719 | 0.001 |
| Proximal to a nearby bifurcation | 6.379 | 4.121-9.874 | <0.001 | 3.897 | 2.487-6.106 | <0.001 |
| MLA<2.51mm^2^ | 10.685 | 3.441-33.172 | <0.001 | 4.131 | 2.341-7.290 | <0.001 |
| AS>64.02% | 7.689 | 2.960-19.971 | <0.001 | 5.574 | 3.337-9.311 | <0.001 |
| RVD | 0.899 | 0.517-1.564 | 0.706 |  |  |  |
| TCFA | 2.650 | 1.520-4.620 | 0.001 | 2.103 | 1.375-3.217 | 0.001 |
| Cholesterol crystals | 1.762 | 1.055-2.942 | 0.030 | 1.673 | 1.142-2.450 | 0.008 |
| Macrophages | 1.571 | 0.383-6.451 | 0.530 |  |  |  |
| Calcification | 0.556 | 0.315-0.980 | 0.042 | 0.912 | 0.513-1.620 | 0.754 |
| Spotty calcium | 0.459 | 0.231-0.913 | 0.026 | 0.611 | 0.332-1.124 | 0.113 |
| Microchannels | 0.948 | 0.323-2.781 | 0.922 |  |  |  |

AS = area stenosis; CI = confidence interval; LAD = left anterior descending artery; LRP = lipid rich plaque; MLA = minimal lumen area; OR = odds ratio; PE = plaque erosion; RVD = reference vessel diameter; TCFA = thin-cap fibroatheroma.

**Supplementary Table 4 Univariate and multivariate analysis of culprit PE in non-LRPs**

| **Variables** | **Univariate analysis** | | | **Multivariate analysis** | | |
| --- | --- | --- | --- | --- | --- | --- |
|  | **OR** | **95%CI** | ***p*-value** | **OR** | **95%CI** | ***p*-value** |
| LAD | 3.261 | 2.401-4.431 | <0.001 | 2.659 | 1.821-3.884 | <0.001 |
| Distance from ostium <40mm | 2.526 | 1.734-3.680 | <0.001 | 1.893 | 1.249-2.869 | 0.003 |
| Proximal to a nearby bifurcation | 2.404 | 1.599-3.613 | <0.001 | 2.863 | 1.866-4.390 | <0.001 |
| MLA<2.51mm^2^ | 4.547 | 3.227-6.406 | <0.001 | 1.729 | 1.123-2.664 | 0.013 |
| AS>64.02% | 9.097 | 6.507-12.719 | <0.001 | 7.029 | 4.672-10.574 | <0.001 |
| RVD | 0.996 | 0.646-1.536 | 0.986 |  |  |  |
| Cholesterol crystals | 2.509 | 1.532-4.111 | <0.001 | 1.587 | 0.858-2.936 | 0.141 |
| Macrophages | 0.764 | 0.521-1.120 | 0.167 |  |  |  |
| Calcification | 0.706 | 0.472-1.055 | 0.089 | 0.493 | 0.267-0.908 | 0.023 |
| Spotty calcium | 0.627 | 0.379-1.038 | 0.070 | 1.005 | 0.501-2.016 | 0.989 |
| Microchannels | 0.640 | 0.427-0.959 | 0.030 | 0.618 | 0.394-0.969 | 0.036 |

AS = area stenosis; CI = confidence interval; LAD = left anterior descending artery; LRP = lipid-rich plaque; MLA = minimal lumen area; OR = odds ratio; PE = plaque erosion; RVD = reference vessel diameter.

**Supplementary Table 5 Univariate and multivariate analysis of culprit PE in female**

| **Variables** | **Univariate analysis** | | | **Multivariate analysis** | | |
| --- | --- | --- | --- | --- | --- | --- |
|  | **OR** | **95%CI** | ***p*-value** | **OR** | **95%CI** | ***p*-value** |
| LAD | 2.814 | 1.840-4.302 | <0.001 | 1.703 | 1.004-2.889 | 0.048 |
| Distance from ostium <40mm | 2.441 | 1.052-5.660 | 0.038 | 1.156 | 0.588-2.271 | 0.674 |
| Proximal to a nearby bifurcation | 2.797 | 1.537-5.091 | 0.001 | 2.792 | 1.648-4.731 | <0.001 |
| MLA<2.51mm^2^ | 2.063 | 1.032-4.127 | 0.041 | 1.482 | 0.696-3.155 | 0.308 |
| AS>64.02% | 2.571 | 0.977-6.765 | 0.056 | 6.153 | 2.709-13.976 | <0.001 |
| RVD | 0.508 | 0.430-0.600 | <0.001 | 0.516 | 0.341-0.780 | 0.002 |
| LRP | 1.198 | 0.775-1.852 | 0.416 |  |  |  |
| TCFA | 1.295 | 0.623-2.693 | 0.489 |  |  |  |
| Cholesterol crystals | 1.373 | 0.704-2.681 | 0.353 |  |  |  |
| Macrophages | 0.862 | 0.528-1.408 | 0.552 |  |  |  |
| Calcification | 1.023 | 0.636-1.645 | 0.925 |  |  |  |
| Spotty calcium | 0.798 | 0.512-1.243 | 0.318 |  |  |  |
| Microchannels | 1.025 | 0.566-1.857 | 0.935 |  |  |  |

AS = area stenosis; CI = confidence interval; LAD = left anterior descending artery; LRP = lipid rich plaque; MLA = minimal lumen area; OR = odds ratio; PE = plaque erosion; RVD = reference vessel diameter; TCFA = thin-cap fibroatheroma.

**Supplementary Table 6 Univariate and multivariate analysis of culprit PE in male**

| **Variables** | **Univariate analysis** | | | **Multivariate analysis** | | |
| --- | --- | --- | --- | --- | --- | --- |
|  | **OR** | **95%CI** | ***p*-value** | **OR** | **95%CI** | ***p*-value** |
| LAD | 3.772 | 2.454-5.798 | <0.001 | 2.428 | 1.714-3.438 | <0.001 |
| Distance from ostium <40mm | 3.585 | 2.030-6.330 | <0.001 | 2.665 | 1.649-4.309 | <0.001 |
| Proximal to a nearby bifurcation | 4.335 | 2.564-7.328 | <0.001 | 3.677 | 2.523-5.361 | <0.001 |
| MLA<2.51mm^2^ | 6.227 | 4.302-9.013 | <0.001 | 2.476 | 1.654-3.707 | <0.001 |
| AS>64.02% | 6.327 | 3.439-11.638 | <0.001 | 4.582 | 3.040-6.904 | <0.001 |
| RVD | 0.915 | 0.635-1.319 | 0.633 |  |  |  |
| LRP | 0.825 | 0.422-1.611 | 0.572 |  |  |  |
| TCFA | 2.685 | 1.384-5.210 | 0.003 | 1.741 | 1.116-2.714 | 0.014 |
| Cholesterol crystals | 1.345 | 0.709-2.549 | 0.364 |  |  |  |
| Macrophages | 1.431 | 0.843-2.428 | 0.184 |  |  |  |
| Calcification | 0.609 | 0.318-1.167 | 0.135 |  |  |  |
| Spotty calcium | 0.507 | 0.240-1.068 | 0.074 | 0.416 | 0.282-0.615 | <0.001 |
| Microchannels | 0.815 | 0.452-1.468 | 0.495 |  |  |  |

AS = area stenosis; CI = confidence interval; LAD = left anterior descending artery; LRP = lipid rich plaque; MLA = minimal lumen area; OR = odds ratio; PE = plaque erosion; RVD = reference vessel diameter; TCFA = thin-cap fibroatheroma.

**Reference**

1. Dai J, Xing L, Jia H, Zhu Y, Zhang S, Hu S, et al. In vivo predictors of plaque erosion in patients with ST-segment elevation myocardial infarction: a clinical, angiographical, and intravascular optical coherence tomography study. *Eur Heart J*. (2018) 39:2077-85. doi: 10.1093/eurheartj/ehy101.
2. Inker LA, Astor BC, Fox CH, Isakova T, Lash JP, Peralta CA, et al. KDOQI US commentary on the 2012 KDIGO clinical practice guideline for the evaluation and management of CKD. *Am J Kidney Dis.* (2014) 63:713-35. doi: 10.1053/j.ajkd.2014.01.416.
3. Tearney GJ, Regar E, Akasaka T, Adriaenssens T, Barlis P, Bezerra HG, et al. Consensus standards for acquisition, measurement, and reporting of intravascular optical coherence tomography studies: A report from the international working group for intravascular optical coherence tomography standardization and validation. *J Am Coll Cardiol.* (2012) 59:1058-72. doi: 10.1016/j.jacc.2011.09.079
4. Kajander OA, Koistinen LS, Eskola M, Huhtala H, Bhindi R, Niemelä K, et al. Feasibility and repeatability of optical coherence tomography measurements of pre-stent thrombus burden in patients with STEMI treated with primary PCI. *Eur Heart J Cardiovasc Imaging*. (2015) 16:96-107. doi: 10.1093/ehjci/jeu175
5. Amabile N, Hammas S, Fradi S, Souteyrand G, Veugeois A, Belle L, et al. Intra-coronary thrombus evolution during acute coronary syndrome: Regression assessment by serial optical coherence tomography analyses. *Eur Heart J Cardiovasc Imaging.* (2015) 16:433-40. doi: 10.1093/ehjci/jeu228
